# Perceptions of neighborhood social environment and drug dependence among incarcerated women and men: a cross-sectional analysis

**DOI:** 10.1186/1747-597X-7-39

**Published:** 2012-09-10

**Authors:** Jessica D Rogers, Megha Ramaswamy, Chin-I Cheng, Kimber Richter, Patricia J Kelly

**Affiliations:** 1 Department of Preventive Medicine and Public Health, University of Kansas School of Medicine, 3901 Rainbow Boulevard, Kansas City, KS, 66160, USA; 2 Department of Mathematics, Central Michigan University, Pearce Hall 214, Mount Pleasant, MI, 48859, USA; 3 School of Nursing, University of Missouri-Kansas City, 2464 Charlotte Street, Kansas City, MO, 64108, USA

**Keywords:** Neighborhood, Drug dependence, Incarceration

## Abstract

**Background:**

Perception of neighborhood social environment can influence an individual’s susceptibility to drug dependence. However, this has never been examined with a jailed sample, where frequent transitions between local jails and disadvantaged neighborhoods are common. Understanding these associations could aid in the design of targeted programs to decrease drug dependence and recidivism among the incarcerated.

**Methods:**

For this study, 596 women and men from three Kansas City jails were surveyed over the course of six months in 2010. Drug dependence was assessed with DSM-IV criteria. Independent variables included fear of one’s neighborhood, perceived level of neighborhood violence, and social capital. All data were self-reported and were analyzed using logistic regression.

**Results:**

Controlling for gender and age, fear of neighborhood violence was associated with increased odds of having drug dependence (OR = 1.27, CI 1.02, 1.58) and a higher level of social capital prior to incarceration was associated with lower odds of drug dependence (OR = 0.65, CI 0.44, 0.96). Mental health problem diagnosis and past year intimate partner violence were significant mediating factors. Gender and race/ethnicity were significant moderating factors between neighborhood disadvantage and drug dependence.

**Conclusions:**

Our study suggests that drug dependence programs for women and men who cycle between jails and communities require both individual- and community-level interventions. To be most effective, programs at the community-level should focus on helping specific groups navigate their communities, as well as address individual health needs associated with drug dependence.

## Background

The majority of women and men in jails are awaiting adjudication or probation [1]. Many are incarcerated for parole violations, and have sentences of one-year terms or less [1]. Once these individuals are released and return home following incarceration, they are often concentrated in a relatively small number of urban centers and neighborhoods that face many challenges, such as high rates of poverty, low rates of health insurance, and inadequate health and social service infrastructures [2]. Half of these previously incarcerated men and women are rearrested in these same neighborhoods within a year [3]. Most return to their neighborhoods after release from jail, some locked in a vicious cycle of incarceration, brief periods of community life, and parole violation or re-arrest on a new offense [4]. High recidivism rates in the United States leads to a “churning” effect – the process of incarcerating, releasing, and re-incarcerating individuals, making transitions between disadvantaged neighborhoods and jail common [5]. Through these mechanisms, incarceration and poor perception of neighborhood social environment (e.g., perceived neighborhood violence and lack of social capital) are inextricably linked.

Incarcerated women and men have higher rates of drug dependence than the general population, owing in large part to the policies of the current ‘war on drugs,’ including mandatory minimum sentences and stiff penalties for street drugs common in poor neighborhoods [6]. A review of drug misuse and dependence among prisoners found that between 10-48% of men and 30-60% of women experienced drug misuse or dependence prior to their incarceration, compared to 4-6% and 2-3% of men and women in the general population, respectively [7,8]. Not only do individuals with criminal justice histories bear a disproportionate burden of drug problems compared to their non-incarcerated counterparts, but they are also often targeted by the criminal justice system rather than being given appropriate drug treatment [9]. Frequent cycling between jails and communities makes it difficult for people with drug dependence problems to gain access to appropriate drug treatment, and perhaps more importantly, to avoid environments and peer groups where drug use is common.

### Theoretical framework

Drug dependence research has traditionally focused on characteristics at the individual- and interpersonal-levels to identify those who are at risk for drug misuse and dependence [10-12]. These traditional predictors include individual, family, and peer variables. While they have been found to be strong predictors for specific groups of people (e.g., Caucasian adolescents) findings do not generalize well to other marginalized groups [13] who are often segregated into the most resource poor areas of towns and cities. Increasing evidence suggests that for groups such as the urban poor, environment (community-level factors) may be a stronger predictor of drug problems than individual-level characteristics [14]. When we look beyond an individual’s home, the broader social and neighborhood environments have been shown to have pervasive effects on substance use and dependence [10].

It is also important to look at how an individual interprets or perceives her or his environment. A study done with adult males comparing narcotic addicts to two control groups (peer and community controls) found clear differences in retrospective perceptions of neighborhood deviance (measured by asking subjects about the frequency of activities such as illegal gambling, violence, prostitution, and drug trafficking in their neighborhood). Addicts had the highest perceptions of neighborhood deviance, and community controls had the lowest [15]. Previous research has argued that it is how key features of the environment are perceived or known that explains the ultimate development of deviant behavior [16].

**Figure 1:**
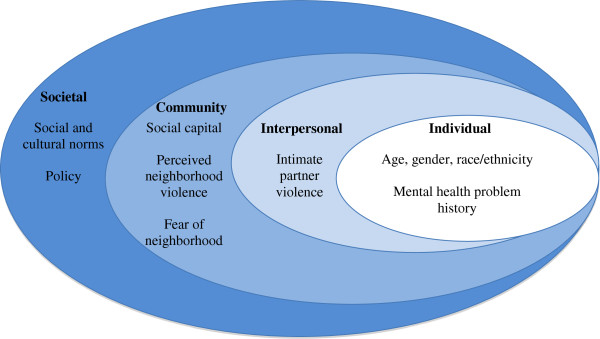
**Social ecological model with measures used in the present study (Adapted from CDC model**[16]**).**

The social ecological model, as adapted by the Centers for Disease Control and Prevention, is a four-level model used to examine the complex interplay between individual, interpersonal, community, and societal factors [17]. This model acknowledges that each of these levels has an impact on the actions that individuals take, and views behavioral patterns as the outcome of interest. The ecological perspective also “implies reciprocal causation between the individual and the environment” [18], an idea that has played out in numerous communities changed by mass incarceration [19]. While many studies have focused on associating individual-level characteristics with drug dependence (which may support a victim-blaming ideology), this study used the social ecological model to examine the community impact on drug dependence among incarcerated women and men (Figure1). 

This study, in particular, focused on the interplay between community-, interpersonal-, and individual-level variables. In this study, we investigated community-level variables as the primary exposures of interest and investigate interpersonal- and individual-level variables as mediators and moderators. All independent variables were chosen based on the hypothesized relationship between neighborhood social environment and drug dependence. Since individuals in U.S. jails are generally incarcerated for less than one year, and many leave as soon as 48 hours after arrest [1], they retain substantial contact with outside communities. Therefore, variables and life conditions *prior to incarceration* may be more related to outcomes than variables unique to an incarcerated environment, and we assessed life conditions accordingly.

At the community-level, for example, we measured perception of neighborhood violence. Published research of non-incarcerated samples also shows that perceived neighborhood violence and level of perceived safety (which we operationalized as fear of neighborhood violence and perceived level of neighborhood violence) are important factors in susceptibility to substance use [13,14]. We also measured social capital, which encompasses community-level factors, such as trust in neighbors and overall assessment of neighborhood prosperity. The literature demonstrates repeatedly that in areas lacking social capital, these types of adverse social conditions may lead to several kinds of deviant behavior, including substance abuse [15,16,20,21].

Drug dependence, of course, has also been associated with many other individual-level characteristics, such as mental health history, intimate partner violence, gender, and race/ethnicity, all of which may interact with community-level variables. For example, mental health problems may act as a barrier to successful recovery from drug problems [22]. Fear and experience of violence also increase the prevalence of depressive symptoms [23], which is a common co-morbidity with substance abuse. This led us to explore mental health problem history as a potential mediator between perceived neighborhood social environment and drug dependence [14]. Drug and alcohol use, in addition to beliefs about drugs and alcohol as coping mechanisms, have also been associated with an increased likelihood or severity of intimate partner violence [24,25]. And neighborhood disorder, that is, a neighborhood lacking the structure necessary to maintain social control and safety, has been linked to increased risk for intimate partner violence in both men and women [26]. Therefore we also treated intimate partner violence as a possible explanatory factor of the complex relationship between perceived neighborhood social environment and drug dependence.

As for moderating variables we hypothesized that there may be important gender and racial/ethnic differences in perceived neighborhood effects on drug dependence. Formerly incarcerated women, in particular, are also forced to navigate their environments in ways that are different than men – balancing obligations to children, sex partners, negotiating housing, and avoiding unique health risks [27-29]. If it is true that women’s and men’s drug use patterns are affected by their environment in different ways as others have shown [13,14], then we could expect the link between perceived neighborhood social environment and drug dependence to also be different for incarcerated women and men.

It is also possible that race/ethnicity might moderate the relationship between neighborhood social environment and drug dependence, since racial minorities are often segregated into the most disadvantaged neighborhoods [30]. Few studies have teased out the race-specific effects of neighborhood social environment on drug use [31]. One such study found that Blacks were more likely to have substance use disorders if they lived in affluent neighborhoods [31], which the authors argued can be explained by race-related stress theory (e.g. more stress related to isolation, and thus drug use, for minorities living in mostly White communities). Because our community in Kansas City is a highly segregated Midwestern city, we argue instead, that the pervasive effects of racial segregation, economic disinvestment in community, and violence [32-34] affect segregated Black communities more than they do other communities which tend to be more racially and economically heterogeneous.

This study ultimately assessed whether there was a relationship between perception of neighborhood social environment prior to incarceration and past year drug dependence among an urban jailed sample of women and men. To our knowledge, the impact of community on health has been understudied among a sample of high-risk women and men who move through disadvantaged communities, jails, and back to communities [19]. Study findings could inform future interventions that address the community-, interpersonal-, and individual-level factors associated with drug dependence in this high-risk group of women and men.

## Methods

This secondary data analysis was part of a larger, cross-sectional study of men and women in jails and their use of and access to health care resources. For this analysis, we used the entire convenience sample of 596 men and women from the parent study [35]. Data collection occurred with participants from three jails in the greater Kansas City metropolitan area over a six-month period in 2010.

### Procedures

Participants were recruited on a continual basis over the course of the study using flyers posted at the facility, as well as word-of-mouth recruitment in each jail housing unit by the special programs coordinator. All English-speaking individuals in the facilities were eligible to participate. In order to participate, individuals had to volunteer and be available for interview at the time of a researcher’s visit. After explaining the study and answering questions, individuals signed informed consent forms, and a face-to-face survey was administered by the interviewer. The University of Kansas City – Missouri Institutional Review Board approved the protocol for this study.

### Dependent variable

Drug dependence, the dependent variable, was assessed using a checklist based on DSM-IV criteria [36]. Participants were asked six questions about use of methamphetamines, PCP, heroin, crack, powdered cocaine, and marijuana in the year before incarceration, including: “In the year before your incarceration, did you need to use more drugs to get the same high as when you first started using?” “Did you need to use more drugs than you wanted to?” “Did you try to cut down drug use, but weren't able to?” “Did drugs play a bigger role in your life than you wanted them to?” “Did drugs cause you to give up or spend less time in school, work, with family or friends, or in recreational activities?” and, “Did you ever keep using drugs even though it made you feel bad physically or emotionally?” If participants answered “yes” to three out of six items, they were classified as “drug-dependent.” The Cronbach’s Alpha for these items was 0.87.

### Independent variables

The independent variables in this study included several indicators of perceived neighborhood social environment at the community-level and variables that we thought would be associated with drug dependence. All collected data pertained to time prior to the current incarceration, since men and women move between jails and community with some frequency [1]. We assessed fear of one’s neighborhood with the following question: “In the neighborhood where you lived before being incarcerated, were you afraid you would be hurt by violence?” [37]. Participants reported whether they were afraid none of the time, a little bit of the time, most of the time, or all the time. Perceptions of neighborhood violence in the six months prior to each person’s incarceration was assessed with five items asking participants if they had heard about a fight in which a weapon was used, a violent argument between neighbors or friends, a gang fight, a robbery or mugging, or a murder (Adult Violence Score, [37] – originally adapted from Perceived Neighborhood Violence Scale, [38]). For this neighborhood violence scale, we computed a summary score across types of violence. A higher summary score indicated a greater perceived level of neighborhood violence. The Cronbach’s Alpha for these items was 0.81.

We measured social capital using a ten-item measure that assessed participants’ perceptions of trustworthiness of neighbors, helpfulness of neighbors, whether the neighborhood had prospered, as well as an overall assessment of neighborhood security [39]. Responses were on a four-point Likert scale of strongly disagree, disagree, agree, or strongly agree. A mean scale score was computed for these ten questions with higher scale scores indicating greater social capital within a neighborhood. The Cronbach’s Alpha for these items was 0.85.

### Mediating and moderating variables

We explored the association of mental health problem history and intimate partner violence as mediating variables with our dependent variable, based either on their association with the outcome of drug dependence in the literature or on a posited relationship.

Mental health problem history was assessed with the question, “Have you ever been told by a doctor, dentist, physician’s assistant, nurse, or nurse practitioner that you had… “Depression? Anxiety? Schizophrenia? Bipolar disease?” If the participant answered “Yes” to any of these questions, they were classified as having a history of mental health problems. The Cronbach’s Alpha for these items was 0.74. We assessed intimate partner violence in the year prior to incarceration by asking participants if a sex partner had physically hurt, insulted or screamed at the participant on a regular basis or fairly often (adapted from Verbal HITS Scale [40]). We condensed all types of intimate partner violence (physical, insulting, screaming) into one question and treated this as a dichotomous variable.

We asked each participant to report his or her gender and race/ethnicity, as potential moderating variables in our analyses.

### Data analysis

Because of the characteristics of our dependent variable in this study, we analyzed the data through the Pearson chi-squared test and a series of logistic regression analyses. The logistic regression analyses enabled us to see if there were significant relationships between independent and dependent variables.

Two additional statistical techniques were also applied to the data analysis processes: the testing of mediating and moderator effects. Mediator variables serve to clarify the nature of the relationship between the independent and dependent variables. The mediating effects were tested through a series of four-step regression analyses suggested by Baron and Kenny [41]. Moderating effects, on the other hand, present when the interaction terms are statistically significant. As suggested by Frazier, Tix, and Barron [42], a two-step logistic regression analysis was adopted to detect the moderating effects. In the first step, both independent and moderator variables were entered into the logistic regression equations predicting drug dependence. In the second step, the interaction terms (testing for moderating effects) were entered into the logistic regression equations. To reduce the multicollinearity issues resulting from the use of interaction terms, the variables in these analyses were standardized.

In the models we ran to test mediating effects, the first 3 steps of the 4-step regression needed to be significant (i.e. the independent variable(s) must be related to the dependent variable). So for the tests we excluded from text and tables in the Results section below, at least one step violated this requirement. Therefore, no mediating effects existed and further data analyses were not conducted. Similarly, we tested the moderating effects for gender and race/ethnicity only when the interaction term of the moderator and the independent variable of interest had a significant relationship with our dependent variable. All analyses controlled for age, and all were conducted in SPSS.

## Results

### Sample characteristics

Participants were on average 34.7 (SD = 10.8) years old (see Table 1). We recruited 290 female participants (48.7%) and 306 male participants (51.3%), in order to have roughly equal comparison groups of women and men (about 10% of correctional facilities are made up of females). Most participants in our sample were Black (N = 249, 42.5%) or White (N = 244, 41.6%). Participants reported 31 months (SD = 47.0) on average spent in jail or prison in their lifetimes. They had been incarcerated for 121.0 (SD = 114.4) days in the past year at the time of the interview.

**Table 1:** Sample characteristics, N = 596

**Variable**	***N***	***Percent***	***Mean***	***SD***
Age			34.7	10.8
Gender				
Male	306	51.3%		
Female	290	48.7%		
Race				
Black	249	42.5%		
White	244	41.6%		
Latino	53	9.0%		
Bi-racial	19	3.2%		
Other	21	3.6%		
Lifetime mental health problem history^1^	284	48.2%		
Past year intimate partner violence	192	36.3%		
Past year drug dependence^2^	229	49.9%		
Fear of neighborhood violence prior to incarceration^3^			1.4	0.9
Perception of level of neighborhood violence prior to incarceration^4^			1.3	1.6
Perceived social capital prior to incarceration^5^			2.6	0.5

Notes:

1. Lifetime diagnosis of depression, anxiety, schizophrenia, or bipolar disease.

2. Based on DMS IV criteria. If participants answered “yes” to three out of six items regarding past year drug use, they were classified as “drug-dependent”.

3. Reported on a scale of 1 to 4, with 4 indicating fear of neighborhood violence all of the time.

4. Based on a summary score of whether participants had heard about five types of violence occurring in their neighborhood. A higher summary score indicates a greater perceived level of neighborhood violence.

5. Reported on a scale of 1 to 4, with 4 indicating high social capital.

Two hundred and eighty-four participants (48.2%) reported a lifetime mental health problem diagnosis. A little over one-third (36.3%) reported experiencing intimate partner violence in the past year.

Half of our valid participants (N = 459) reported past year drug dependence (N = 229, 49.89%), with a statistically significant difference between men and women. Female drug dependence was 55.17% (N = 128), and male drug dependence was 44.49% (N = 101) (*χ*2 = 5.23, *d.f.* = 1, *p* = 0.01).

Participants reported living for an average of over seven years in their neighborhoods prior to incarceration. On a scale of 1 to 4, with 4 indicating fear of neighborhood violence all of the time, participants had a mean score of 1.4 (SD = 0.9) on a fear of neighborhood violence scale. Participants reported seeing at least one type of crime in their neighborhood in the six months prior to incarceration (mean = 1.3, SD =1.6). Finally, on a scale of 1 to 4, with 4 indicating a higher level of social capital, participants had an average score of 2.6 on a perception of neighborhood social capital scale.

### Test of mediating effects

As described earlier, the test of mediating effects required a four-step testing procedure. In these analyses, three mediating effects were confirmed. As shown in Table 2, there was a positive relationship between fear of neighborhood and drug dependence (Step 1, *B* = 0.24, *OR* = 1.27, Wald *χ*^2^ = 4.67, *d.f.* = 1, *p* = 0.03). When fear of neighborhood increases one unit, the odds for drug dependence can be predicted to increase by a factor of around 1.27 times. In other words, more fear of neighborhood prior to incarceration was associated with past year drug dependence. While controlling for age and gender, the relationship between drug dependence and fear of neighborhood was no longer statistically significant after mental health problem history was taken into account (Step 4^a^, *B* = 0.17, Wald *χ*^2^ = 2.32, *d.f.* = 1, *p* = 0.13). According to the suggestions from Soble [43] we further confirmed that the decrease was a result of a full mediating effect. 

**Table 2:** **The logistic regression results of the mediating effect of mental health problem history**^**a**^** and intimate partner violence**^**b**^** to the relationship between drug dependence and fear of neighborhood**

**Step and variable**	***B***	***S.E.***	***Wald***	***OR***	***p***	**Dependent**
Step 1						Drug dependence
Age	0.02	0.01	2.35	1.02	0.13	
Gender	−0.45	0.20	5.74	0.64	0.02	
Fear of neighborhood	0.24	0.11	4.67	1.27	0.03	
Constant	−0.19	0.47	0.16	0.83	0.69	
Step 2^a^						Mental health problem history
Age	0.01	0.01	0.05	1.00	0.82	
Gender	−1.00	0.18	31.35	0.37	< 0.001	
Fear of neighborhood	0.37	0.11	12.33	1.45	< 0.001	
Constant	0.78	0.42	3.53	2.18	0.06	
Step 3^a^						Drug dependence
Age	0.02	0.01	3.27	1.02	0.07	
Gender	−0.24	0.20	1.51	0.78	0.22	
Mental health problem history	0.90	0.20	20.51	2.47	< 0.001	
Constant	−0.71	0.47	2.29	0.49	0.13	
Step 4^a^						Drug dependence
Age	0.02	0.01	3.12	1.02	0.08	
Gender	−0.30	0.21	2.11	0.74	0.15	
Fear of neighborhood	0.17	0.11	2.32	1.19	0.13	
Mental health problem history	0.87	0.21	17.89	2.38	< 0.001	
Constant	−0.87	0.51	2.87	0.42	0.09	
Step 2^b^						Intimate partner violence
Age	0.01	0.01	0.25	1.01	0.62	
Gender	−0.87	0.20	19.97	0.42	< 0.001	
Fear of neighborhood	0.36	0.11	11.23	1.43	0.001	
Constant	−0.01	0.44	0.01	0.99	0.98	
Step 3^b^						Drug dependence
Age	0.01	0.01	2.14	1.02	0.14	
Gender	−0.21	0.21	0.97	0.82	0.33	
Intimate partner violence	0.81	0.21	14.49	2.25	< 0.001	
Constant	−0.49	0.47	1.07	0.61	0.30	
Step 4^b^						Drug dependence
Age	0.02	0.01	2.02	1.02	0.16	
Gender	−0.24	0.21	1.24	0.79	0.27	
Fear of neighborhood	0.21	0.12	3.08	1.24	0.08	
Intimate partner violence	0.79	0.22	12.71	2.21	< 0.001	
Constant	−0.75	0.51	2.17	0.47	0.14	

Note: Age and gender are control variables. *B*s were tested through the Wald *χ*^2^ statistic with *d.f.* = 1. ^a^: The tests of mediating effects caused by mental health problem history. ^b^: The tests of mediating effects caused by intimate partner violence.

In Table 2, we showed that while controlling for age and gender, the relationship between drug dependence and fear of neighborhood (Step 1, *B* = 0.24, Wald *χ*^2^ = 4.67, *d.f.* = 1, *p* = 0.03) also became statistically non-significant after intimate partner violence was taken into account (Step 4^b^, *B* = 0.21, Wald *χ*^2^ = 3.08, *d.f.* = 1, *p* = 0.08).

As indicated in Table 3, there was a negative relationship between social capital and drug dependence (Step 1, *B* = −0.43, *OR* = 0.65, Wald *χ*^2^ = 4.51, *d.f.* = 1, *p* = 0.03). When social capital increases one unit, the odds for drug dependence can be predicted to decrease by a factor of around 0.65 times. In other words, more social capital prior to incarceration was associated with less past year drug dependence. While controlling for age and gender, the relationship between drug dependence and social capital was no longer statistically significant after mental health problem history was taken into account (Step 4, *B* = −0.38, Wald *χ*^2^ = 3.32, *d.f.* = 1, *p* = 0.07).

**Table 3:** The logistic regression results of the mediating effect of mental health problem history to the relationship between drug dependence and social capital

**Step and variable**	***B***	***S.E.***	***Wald***	***OR***	***p***	**Dependent**
Step 1						Drug dependence
Age	0.02	0.01	2.95	1.02	0.09	
Gender	−0.41	0.19	4.73	0.66	0.03	
Social capital	−0.43	0.20	4.51	0.65	0.03	
Constant	1.16	0.65	3.22	3.20	0.07	
Step 2						Mental health problem history
Age	0.01	0.01	0.11	1.00	0.74	
Gender	−1.08	0.17	38.75	0.34	< 0.001	
Social capital	−0.41	0.19	4.85	0.66	0.03	
Constant	2.50	0.59	18.22	12.23	< 0.001	
Step 3						Drug dependence
Age	0.02	0.01	3.27	1.02	0.07	
Gender	−0.24	0.20	1.51	0.78	0.22	
Mental health problem history	0.90	0.20	20.51	2.47	< 0.001	
Constant	−0.71	0.47	2.29	0.49	0.13	
Step 4						Drug dependence
Age	0.02	0.01	3.79	1.02	0.05	
Gender	−0.24	0.20	1.40	0.79	0.24	
Social capital	−0.38	0.21	3.32	0.68	0.07	
Mental health problem history	0.85	0.20	17.95	2.34	< 0.001	
Constant	0.22	0.69	0.10	1.24	0.75	

Note: Age and gender are control variables. *B*s were tested through the Wald *χ*^2^ statistic with *d.f.* = 1.

### Test of moderating effects

**Figure 2:**
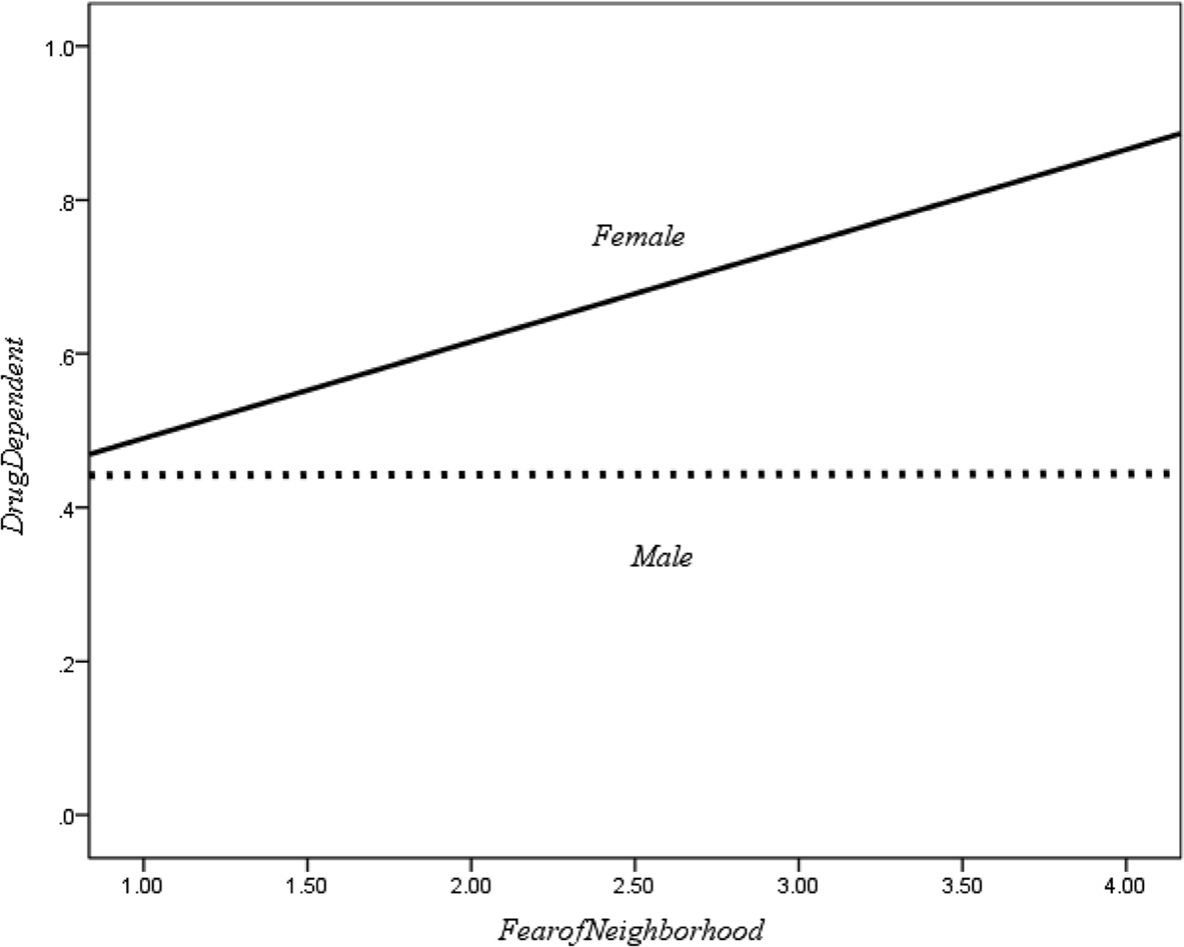
**Moderator effect of gender to the drug dependence-fear of neighborhoodrelationship.**

Although gender was a control variable in the three mediating effects tests, the results indicated that gender was significantly related to the dependent variable and that it might have altered interpretation of the study results. Therefore, gender was selected as a moderator in our study. After running a logistic regression test of the moderating effect of gender to the relationship between drug dependence and fear of neighborhood, we found that the interaction term of gender and fear of neighborhood had a significant relationship with drug dependence (*B* = −0.56, Wald *χ*^2^ = 5.42, *d.f.* = 1, *p* = 0.02). Figure 2, which summarized the relationship between fear of neighborhood and drug dependence, indicated that past year drug dependence is positively related to fear of neighborhood prior to incarceration for the female group, while no relationship was indicated for the male group.

**Figure 3:**
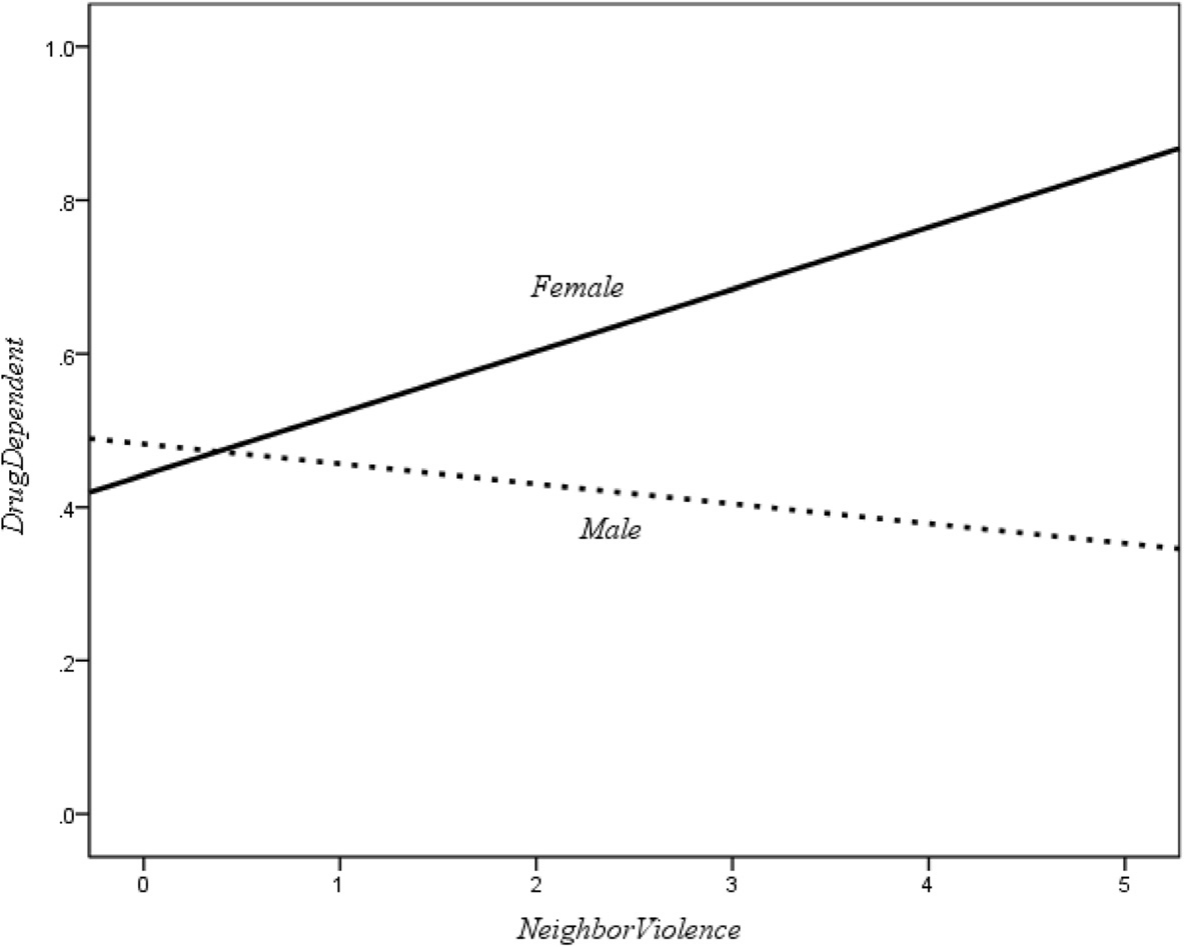
Moderator effect of gender to the drug dependence-perceived neighborhood violence relationship.

The interaction term of gender and perceived neighborhood violence also had a significant relationship with drug dependence (*B* = −.45, Wald *χ*^2^ = 12.08, *d.f.* = 1, *p* < 0.01). Figure 3 indicated that drug dependence was positively related to perceived neighborhood violence prior to incarceration for the female group, while no significant relationship was indicated for males.

**Figure 4:**
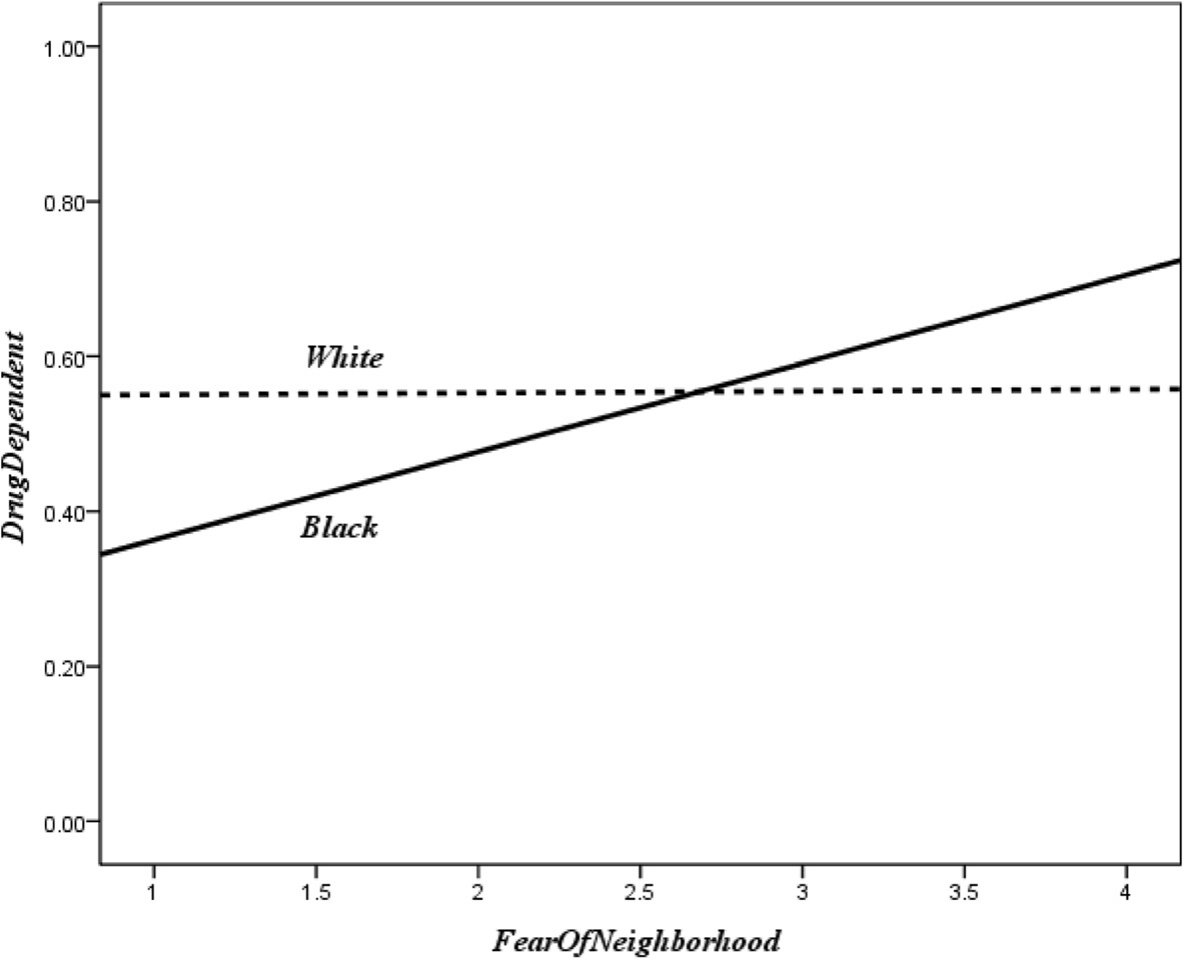
Moderator effect of race to the drug dependence-fear of neighborhood relationship.

The interaction term for race/ethnicity (Black vs. White, since these groups comprised the majority of our sample) and fear of neighborhood had a significant relationship with drug dependence (*B* = 0.45, Wald *χ*^2^ = 4.38, *d.f.* = 1, *p* = 0.04). Figure 4 summarized the two relationship patterns due to racial/ethnic difference, which indicated that drug dependence was positively related to fear of neighborhood for Blacks, while no relationship was indicated for the Whites.

### Test of mediating effects for females and males

Due to the moderating effect of gender on the relationship between independent and dependent variables of interest, we split the dataset according to participants’ gender and ran a series of mediating effects tests. Among these tests, three mediating effects were confirmed. As indicated in Table 4, for the female group, while controlling for age, the relationship between drug dependence and fear of neighborhood (Step 1, *B* = 0.56, Wald *χ*^2^ = 9.00, *d.f.* = 1, *p* = 0.01) decreased after mental health was taken into account (Step 4^a^, *B* = 0.48, Wald *χ*^2^ = 6.47, *d.f.* = 1, *p* = 0.01). However, for the male group, drug dependence was not related to fear of neighborhood; therefore, no mediating test was needed.

**Table 4:** **The logistic regression results of the mediating effect of mental health problem history**^**a**^** and intimate partner violence**^**b**^** to the relationship between drug dependence and fear of neighborhood by gender**

**Gender**	**Step and variable**	**B**	**S.E.**	**Wald**	**OR**	**p**	**Dependent**
Female	Step 1						Drug dependence
	Age	0.01	0.02	0.60	1.01	0.44	
	Fear of neighborhood	0.56	0.19	9.00	1.75	0.01	
	Constant	−1.01	0.59	2.97	0.37	0.09	
	Step 2^a^						Mental health problem history
	Age	0.01	0.01	0.45	1.01	0.50	
	Fear of neighborhood	0.53	0.18	8.83	1.71	0.01	
	Constant	0.69	0.52	1.75	0.50	0.19	
	Step 3^a^						Drug dependence
	Age	0.01	0.02	0.82	1.01	0.37	
	Mental health problem history	1.13	0.29	15.52	3.08	< 0.001	
	Constant	−0.97	0.55	3.13	0.38	0.08	
	Step 4^a^						Drug dependence
	Age	0.01	0.02	0.61	1.01	0.44	
	Fear of neighborhood	0.48	0.19	6.47	1.61	0.01	
	Mental health problem history	1.06	0.31	11.97	2.88	0.001	
	Constant	−1.55	0.63	6.07	0.21	0.01	
	Step 2^b^						Intimate partner violence
	Age	0.01	0.01	0.10	1.00	0.76	
	Fear of neighborhood	0.52	0.16	10.88	1.68	< 0.001	
	Constant	−1.10	0.51	4.66	0.33	0.03	
	Step 3^b^						Drug dependence
	Age	0.01	0.02	0.28	1.01	0.60	
	Intimate partner violence	1.01	0.28	13.03	2.73	< 0.001	
	Constant	−0.57	0.52	1.18	0.57	0.28	
	Step 4^b^						Drug dependence
	Age	0.01	0.02	0.32	1.01	0.57	
	Fear of neighborhood	0.45	0.19	5.35	1.56	0.02	
	Intimate partner violence	0.93	0.30	9.45	2.54	0.002	
	Constant	−1.19	0.60	3.92	0.30	0.05	

Note: Age is the control variable. *B*s were tested through the Wald *χ*^2^ statistic with *d.f.* = 1. ^a^: The tests of mediating effects caused by mental health problem history. ^b^: The tests of mediating effects caused by intimate partner violence. The test results for the male group were omitted, as the results were not significant.

Table 4 showed that for females, while controlling for age, the relationship between drug dependence and fear of neighborhood (Step 1, *B* = 0.56, Wald *χ*^2^ = 9.00, *d.f.* = 1, *p* = 0.01) also decreased after intimate partner violence was taken into account (Step 4^b^, *B* = 0.45, Wald *χ*^2^ = 5.35, *d.f.* = 1, *p* = 0.02).

Mental health problem history also mediated the relationship between drug dependence and social capital. As indicated in Table 5, for females, while controlling for age, the relationship between drug dependence and social capital (Step 1, *B* = −0.61, Wald *χ*^2^ = 4.77, *d.f.* = 1, *p* = 0.03) decreased after mental health problem history was taken into account (Step 4, *B* = −0.59, Wald *χ*^2^ = 4.24, *d.f.* = 1, *p* = 0.04).

**Table 5:** The logistic regression results of the mediating effect of mental health to the relationship between drug dependence and social capital by gender

**Gender**	**Step and variable**	***B***	***S.E.***	***Wald***	***OR***	***p***	**Dependent**
Female	Step 1						Drug dependence
	Age	0.02	0.01	1.06	1.02	0.30	
	Social capital	−0.61	0.28	4.77	0.54	0.03	
	Constant	1.24	0.85	2.12	3.46	0.15	
	Step 2						Mental health problem history
	Age	0.01	0.01	0.32	1.01	0.57	
	Social capital	−0.33	0.25	1.76	0.72	0.19	
	Constant	1.06	0.76	1.94	2.90	0.16	
	Step 3						Drug dependence
	Age	0.01	0.02	0.82	1.01	0.37	
	Mental health problem history	1.13	0.29	15.52	3.08	< 0.001	
	Constant	−0.97	0.55	3.13	0.38	0.08	
	Step 4						Drug dependence
	Age	0.02	0.02	1.13	1.02	0.29	
	Social capital	−0.59	0.29	4.24	0.55	0.04	
	Mental health problem history	1.07	0.29	13.60	2.91	< 0.001	
	Constant	0.49	0.90	0.30	1.63	0.59	

Note: Age is the control variable. *B*s were tested through the Wald *χ*^2^ statistic with *d.f.* = 1. The test results for the male group were omitted, as the results were not significant.

No other moderating effects were found for race/ethnicity. And after dividing the sample into two groups (Black vs. White), no further significant mediating effects were found.

## Discussion

Our findings suggest that drug problems are not an isolated or random occurrence, but rather they are related to individual health histories, interpersonal relationships, and perception of the broader social environment. This finding is congruent with the social ecological model. The perceived components of the neighborhood environment (community-level variables) are associated with an individual’s vulnerability to adult drug dependence. Many factors in the geographic neighborhood are outside an individual’s control [10]. A person’s choice of neighborhood is often dictated by circumstances rather than personal desire. A person’s ability to avoid violence at the neighborhood level and within interpersonal relationships may also affect her or his ability to avoid drug use. Interestingly, our data indicated that females were both more significantly and more directly affected by their neighborhood environment compared to men. Finally, mental health problem history mediated the relationship between perceived neighborhood social environment and drug dependence within our sample, suggesting that neighborhood conditions may be associated with mental health problems [23], and thus, drug problems.

Our finding that fear of neighborhood violence is a significant factor associated with drug dependence is consistent with published research of non-incarcerated samples, which shows that level of perceived safety is an important factor in susceptibility to substance use [13,14]. It should also be noted that for a drug-using sample or group of people who live in places where drug use is prevalent, social networks may be influenced by common perceptions about neighborhoods, including the feeling that neighborhoods are unsafe [44]. Other non-drug using members of the community or those outside of drug-using networks may not necessarily feel similarly, given lack of involvement in drug-using networks.

We also found that the relationship between fear of neighborhood and drug dependence was mediated by both mental health problem diagnosis and past year intimate partner violence. This also agrees with previous research, such as large population-based studies, which have shown the high prevalence of mental health problems among illicit drug users, as described above [45,46]. Due to the cross-sectional design of our study, it is difficult to suggest whether fear of neighborhood directly influences mental health problems, and subsequently drug dependence. However, fear of neighborhood certainly does not alleviate existing problems with depression and anxiety, thus failing to disrupt the simultaneous battle for many with both mental health and drug problems. Intimate partner violence, as a mediator, however, is likely to influence the perception that one’s neighborhood is a scary and dangerous place, leading to poor coping behaviors, such as drug use [25]. But again, our cross-sectional study design prevented us from determining the exact mechanisms of these relationships.

Our analyses showed that social capital was a significant factor associated with drug dependence. Social capital encompasses environmental factors such as trust in neighbors and overall assessment of neighborhood. Incarcerated individuals with higher social capital, regardless of age or gender, were found to be less likely to be drug dependent adults. This finding reflects the literature that demonstrates that adverse social conditions in disadvantaged neighborhoods may lead to several types of deviant behavior, including substance abuse [15,16,20,21]. Similar to our finding about fear of neighborhood violence, mental health problem history mediated the relationship between perceived social capital and drug dependence.

When examining gender differences, we found that perception of neighborhood environment may affect women more than men, even when taking other factors, such as mental health problem history, into account. We hypothesized that this finding may have to do with how women are forced to navigate their social environments, for example, participation in their children’s health care, education, and welfare. In some ways, women may lead lives that rely on community involvement, and lacking in social capital to navigate communities could be a detriment to health, including drug use [27-29]. We also found that Blacks may be more affected by neighborhood social environment compared to Whites, when it comes to drug dependence. Neighborhood social environment, which could be a marker for community violence, lack of social cohesion, trust, and lack of economic development, may have greater effects on segregated communities and on the minorities who reside in those communities. Further research is needed to flesh out this relationship.

### Strengths and limitations

In this study we looked at a well-known problem, drug dependence, through a new lens. By examining how drug dependence is affected by neighborhood social environment among a sample that cycles between jails and these neighborhoods, we gain insight that can be applied to the problem of incarceration, as well as that of drug dependence. Framing our study within the social ecological model allowed us to examine the problem as it truly is – at the community, interpersonal, and individual level.

A major limitation of this study was recruitment of a relatively small convenience sample. Due to the way the sample was obtained, we cannot be sure that participants’ experiences are representative of the incarcerated women and men who did not choose to volunteer for this study. We also recruited an English-speaking sample. However, the demographics (including report of Latino race/ethnicity) of our sample did reflect those of inmates who did not participate in the study but were incarcerated in Kansas City jails. Results also may not be valid or generalizable outside of the Kansas City Metropolitan area, or to a non-incarcerated sample. A second limitation is that the study relied on self-report of drug dependence, and due to the retrospective nature of study questions, the data may be subject to recall bias. However, we used DSM-IV criteria to judge this self-reported data as it relates to drug dependence categorization. A third limitation of the study is that we used single-item measures to assess mental health problem history and fear of neighborhood. Though these items were associated with our dependent variable, they may not be as reliable as more robust measures. Fourth, although some geographic information regarding participants’ neighborhoods was collected, the self-reported perceptions of neighborhood environment were not compared with external data sources, such as arrest rates or community-level indicators of neighborhood social environment, such as unemployment rates, high school attainment at the community-level, or poverty rates. Finally, this study does not establish a temporal relationship between perceptions of neighborhood environment and drug dependence. It is possible that participants began using substances before moving into the neighborhood they perceived as disadvantaged.

### Areas for future research and policy

Future research should focus on differences in the ways that males and females cope with a negative social environment, in order to better understand the effects that neighborhoods have on women and men, especially when taking into account interpersonal- and individual-level factors. We should also attempt to find the most effective ways to increase social networks and capital for females in disadvantaged communities so that they can better cope with fears about violence in their neighborhoods and within intimate relationships. Finally, such interventions must go hand in hand with better individual-level treatment options for mental health problems, which are important individual-level predictors of drug problems. Aside from the influence of neighborhood social environment, policies relating to *who* we incarcerate and how we rehabilitate them (drug users and individuals with mental health problem histories [1,9,19]) must be revisited.

## Conclusion

We found that poor perception of neighborhood social environment – particularly in the form of fear of neighborhood and low perceived social capital – was associated with drug dependence among incarcerated women and men. However, the effects of the neighborhood environment appeared to be different for females and males, with females being more strongly affected by their neighborhood. Finally, mental health problem history maintains an important association with drug dependence, alongside other interpersonal factors, such as violence in the home. Our study suggested that both individual- and community-level interventions are required in disadvantaged neighborhoods to decrease drug dependence among women and men who cycle between these neighborhoods and local jails. Interventions that address drug dependence for this group of women and men that cycle between disadvantaged neighborhoods and jails must be as multifactorial as the causes of drug dependence. Substance deterrence programs in such neighborhoods would include, for example, a focus on helping females make connections within their own community. Programs must also address this high-risk group’s mental health and interpersonal violence histories. Finally, broader social policies – in housing, health care, education, and employment – must move beyond the purely punitive approaches of the criminal justice system and refocus efforts on community development and individual rehabilitation.

## Competing interests

The authors declare that they have no competing interests.

## Authors’ contributions

JDR made contributions to the conception and design of analytic plan and drafted and revised the manuscript. MR made contributions in acquisition of data, to the conception and design of analytic plan, and revised the manuscript. CC performed all statistical analyses. KR revised the manuscript. PJK made contributions in acquisition of data and revised the manuscript. All authors read and approved the final manuscript.
